# Biomarkers of Immunotoxicity for Environmental and Public Health Research

**DOI:** 10.3390/ijerph8051388

**Published:** 2011-05-06

**Authors:** Paurene Duramad, Nina T. Holland

**Affiliations:** 1 Genentech, Inc., 1 DNA Way MS #59, South San Francisco, CA 94080, USA; E-Mail: paurened@gene.com; 2 School of Public Health, University of California, Berkeley, 733 University Hall, Berkeley, CA 94720-7360, USA

**Keywords:** asthma, COPD, biomarker validation, immunoinformatics, Luminex, immunome, immunotoxicity

## Abstract

The immune response plays an important role in the pathophysiology of numerous diseases including asthma, autoimmunity and cancer. Application of biomarkers of immunotoxicity in epidemiology studies and human clinical trials can improve our understanding of the mechanisms that underlie the associations between environmental exposures and development of these immune-mediated diseases. Immunological biomarkers currently used in environmental health studies include detection of key components of innate and adaptive immunity (e.g., complement, immunoglobulin and cell subsets) as well as functional responses and activation of key immune cells. The use of high-throughput assays, including flow cytometry, Luminex, and Multi-spot cytokine detection methods can further provide quantitative analysis of immune effects. Due to the complexity and redundancy of the immune response, an integrated assessment of several components of the immune responses is needed. The rapidly expanding field of immunoinformatics will also aid in the synthesis of the vast amount of data being generated. This review discusses and provides examples of how the identification and development of immunological biomarkers for use in studies of environmental exposures and immune-mediated disorders can be achieved.

## 1. Introduction

The field of immunotoxicology has rapidly expanded and the main drivers for this development include recognition that environmental chemicals can alter immune response and function, increase in immune-mediated diseases (asthma, allergies, type 1 diabetes, rheumatoid arthritis, *etc.*) [1–4], and recognition that the immune system plays an important role in the pathophysiology of other disease states such as cancer [1] and atherosclerosis [2–4]. A pro-inflammatory immune response contributes to tissue and organ damage and is a common factor in many auto-immune diseases (e.g., Type 1 diabetes, rheumatoid arthritis, systemic lupus erythermatosus) and other disorders (e.g., promotion of atherosclerotic plaques in cardiovascular disease). Conversely, the lack of an appropriate inflammatory immune response contributes to lowered immune surveillance and the progression of tumors and cancers. A thorough understanding of the role of the immune response in the pathophysiology of these diseases is important to identify efficacious therapies and effective interventions. Further, the prevention of these diseases can be aided when specific and sensitive biomarkers, particularly ones that precede clinical onset of these diseases, are identified.

A biomarker is defined as “a characteristic that is evaluated as an indicator of normal biological or pathogenic processes, or a pharmacological response to a therapeutic intervention [5,6].” Biomarkers have been used for many years in toxicology and risk assessment and are often classified in terms of biomarkers of exposure, effect, and susceptibility [7]. However, categories based on intended roles and applications (e.g., disease, efficacy, mechanism, pharmacodynamic, and target) provide a useful classification system (see Baker *et al.* 2005 [8]). Disease-related biomarkers are mostly used for monitoring disease causality, progression, and susceptibility, and, to some extent, to identify strategies for patient stratification [9]. Both regulatory agencies and industry are keen to identify biomarkers that will aid in the early detection of toxicities [6,7,10,11].

## 2. Key Considerations for Developing Biomarkers of Immunotoxicity

**Figure 1.**
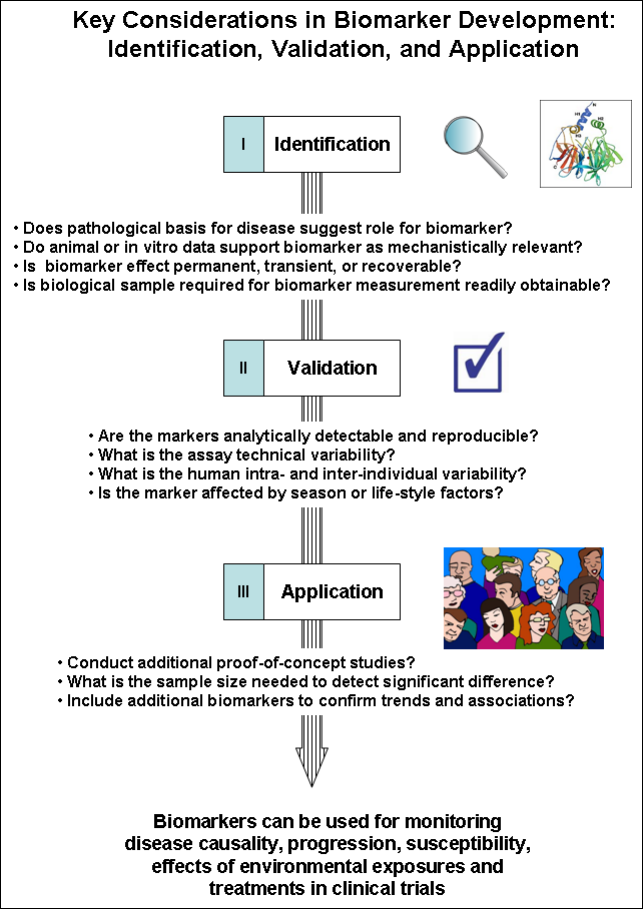
Key considerations in biomarker development: identification, validation, and application.

Development of disease biomarkers broadly involves the three stages of identification, validation, and application (see Figure 1) and key elements of the process include (1) identifying biomarkers that can establish relevance (*i.e.*, related to the disease of exposure of interest), (2) strong, mechanistic molecular or biochemical basis in the pathophysiology of a disease, (3) sensitivity and specificity to treatment or exposure, (4) reliability (reproducibility, accuracy, precision, robustness), (5) practicality (level of assay invasiveness), and (6) simplicity in use and application [12,13]. Rarely does one biomarker meet all six requirements, however early consideration of these parameters in properly-designed and statistically powered studies can improve the final predictive value of biomarkers [14].

Since the immune system is composed of multiple organs (e.g., bone marrow, thymus, spleen and lymph nodes) and an appropriate immune response involves the interaction of multiple cell types (e.g., dendritic, B, and T-helper cells) and pleiotropic components (e.g., immunoglobulin and cytokines) it is a challenge to identify a key parameter to develop as a biomarker. Immunotoxic effects are commonly categorized into one of four categories: immunosuppression (reduction in efficacy or activation of immune system), immunostimulation (general enhanced immune response), hypersensitivity (specific immunostimulatory response mediated by immunoglobulins or T-cells), and autoimmunity (immune response against self). Immunotoxicity refers to any adverse effect on the structure or function of innate and adaptive immunity (see excellent references on this topic [15–18]).

The most common immune markers and sample types are summarized in Table 1. For example, cell-surface markers and antibodies are commonly used to evaluate the status of the immune system and can be detected using whole blood or serum and plasma by a multitude of methods. Although blood collection is an invasive procedure, subjects are accustomed to blood draws. However, if the same biomarker can be detected in samples collected using a non-invasive procedure, such as saliva or urine, these could then be prioritized for development, particularly for studies intended for pediatric populations. Efforts to develop non-invasive collection methods include analysis of immune components in saliva [19,20] or induced sputum [21] and exhaled breath condensate [22].

## 3. Sample Collection and Analytical Methods

Biomarker studies require processing and storage of numerous biological samples with the goals of obtaining a large amount of information and minimizing future research costs. An efficient study design includes provisions for processing of the original samples, such as separation of various components (e.g., serum, plasma, clot *etc*.), stabilization, cryopreservation, DNA isolation, and preparation of specimens for exposure assessment [23]. Standard operating procedures and quality control plans help to protect sample quality and to assure validity of the biomarker data. Data validity can also be affected by the sample type used. For example, proteomic profiles reportedly differ between serum and plasma samples [24] with less reproducibility observed with serum samples [25–27]. Cytokine levels may be lower in serum than in stimulated whole blood cultures [28,29]. The types of blood collection tubes (proteins adsorb to different materials) or anti-coagulants used (heparin and EDTA have different mechanisms for the prevention of clotting) also contribute to variability in the data obtained [30]. Also, the effect of transportation and storage of biological material must be examined very closely as the assay can be time- and temperature-sensitive. These factors, in addition to the assay and method variables listed above, can affect the precision of the measurement. Any significant contributor can then be controlled for in the large-scale epidemiology studies.

Employment of high-throughput methods in large-scale epidemiology studies provide a number of advantages for study designs that involve the collection and timely analysis of numerous clinical samples. Flow cytometry has emerged as a powerful tool for quantitative, single-cell analysis of both surface markers and intracellular antigens. This platform can now be used to measure intracellular signaling cascades and phosphorylation events and are employed extensively in high-throughput drug screening. Multiplex detection of cytokines allows the simultaneous measurement of multiple cytokines in a sample [31]. These platforms increase the efficiency of measuring the cytokines while reducing the serum sample volumes required for the testing, thus replacing the more traditional ELISA-based approach. Compared to Cytokine Bead Array (CBA), Luminex kits were found to be highly reproducible and reliable [32]. Increased standardization between laboratories represents another challenge in the application of immune biomarkers. However, this is one that can be remedied with increased collaboration and exchange of information during which protocols and methods are shared across laboratories and the reproducibility of immune marker detection can be determined before the samples are analyzed. Variability in parameters measured by flow cytometry attributable to subjective gating and/or determination of positive *versus* negative events can be reduced by distributing templates for acquisition and data analysis among the sites involved in analysis [33].

## 4. Case Studies: Asthma and Chronic Obstructive Pulmonary Disease (COPD)

Application of these methods will be illustrated with examples from the field of asthma and chronic obstructive pulmonary disease (COPD), two of the most common disorders of the airways. In both cases, airway obstruction is the result of chronic inflammation and the infiltration of pro-inflammatory cells and mediators [34,35]. However, there are some noteworthy differences in the histopathology and the immune cells recruited for these two diseases [36]. The immune profiles of these diseases are summarized in Table 2 and, although there is a strong association between these immunologic endpoints and disease status, the predictive value of these endpoints are still under investigation.

Bronchial biopsies from asthmatic patients reveal an infilitration of eosinophils, activated mucosal mast and T cells whereas in COPD eosinophils are largely absent but neutrophils are present in large numbers [46]. Also, in COPD, there is also an imbalance of the CD4+/CD8+ T-lymphocyte ratio in the lungs with CD8 predominating [43]. The inflammatory state of the lung is thought to be maintained through recruitment of macrophages and lymphocytes [34]. Increased expression of chemokine receptor CXCR3 on macrophages, and its ligand CXCL10 has been observed in patients with COPD [47]. In support of these findings, Costa *et al*. reported that in addition to CXCR3, chemokine receptors CXCL9, CXCL10, and CXCL11 were elevated in COPD, compared to non-smokers [42]. The levels of inflammatory cytokines (IL-6, IL-8, and TNF-α) are also elevated in COPD [44]. The cumulative data available for this disease is in part due to the extensive validation efforts by researchers to standardize sample collection methods and biomarker endpoints analysis [48].

Asthma is characterized by chronic inflammation in the airways and the presence of a predominance of CD4^+^ T-helper 2 cells that secrete IL-4, IL-5, and IL-13 cytokines [49,50]. Th2 cells contribute to the immunopathogenesis of asthma by recruiting eosinophils and mast cells to the  airways [51,52] and by inducing B-cells to produce immunoglobulin E antibodies [53]. Increased levels of IFN-γ also have been reported in cases of severe asthma that could involve CD8^+^ T cells [54]. In childhood, a major risk factor for the development of persistent asthma is atopy, which is defined by the presence of IgE to common inhalant allergens such as house dust mite [55]. Polymorphisms in CD14, a membrane receptor for bacterial components, have been linked to atopy [56]. In a comprehensive study by Heaton *et al.* [38], multiple immune markers were used to differentiate between various airway disease phenotypes in children. The authors reported that atopic children were more likely to have increased T-helper 2 (Th2) cytokines such as interleukin IL-4, IL-5, IL-13 whereas children with bronchial hyper-reactivity were more likely to have elevated IFN-γ, a Th1 cytokine [38]. The associations of Th1/Th2 are not consistent for all allergic disorders. For example, Kaneko *et al.* [57] reports that atopic dermatitis (AD) is associated with increased IL-4 Th2 cells, whereas Machura *et al.* [58] report that children with AD have significantly lower IL-4 Th2 cells and TNF-α Th1 cells and, therefore, no distinct bias towards Th1 or Th2 profiles.

Hollams *et al.* (2009) [59] sought to identify biomarkers associated with asthma phenotypes in teenagers, particularly atopic asthma, and to identify markers that aid in discriminating between atopic subjects at high *versus* low risk of asthma. In a cohort of 1380 14-year olds, clinical history as well as measurement of circulating and/or inflammatory markers (e.g., eosinophils, IgE, cytokine measurements) and *in vitro* innate and adaptive immune functions (e.g., house dust mite (HDM) T-cell responses) were evaluated. HDM-induced cytokine expression of IL-5, IL-9, IL-10, IL-13, and IFN-γ were significantly elevated in teens with asthma. Due to the redundancy of the immune system, for example IL-5, IL-9 and IL-13 contribute to the Th2 response and generation of IgE, therefore it is important to examine changes in several cytokines simultaneously rather than in isolation.

## 5. Emerging Methods

Advances in technology have introduced a variety of “omic” approaches to study human diseases and identify new biomarkers [60]. Interrogation of DNA (genomics) reflects genetic variability, mRNA (also genomics, sometimes called transcriptomics) displays changes in gene expression, proteins (proteomics) represent cellular and enzymatic changes (proteomics), and metabolites (metabonomics) highlight the physiological endpoints [9]. Toxicogenomics, the identification of specific gene expression profiles in biological systems associated with xenobiotic exposure, is increasingly being applied in immunotoxicity assessments [61]. For example, children with the TGF-β1-509TT genotype are at increased risk of asthma when they are exposed to maternal smoking *in utero* or to traffic-related emissions [62]. In immunotoxicology studies, microarrays have been used mainly in drug development to model pharmacodynamic effects of pharmaceuticals [63]. Multiparameter flow cytometry can also provide insight into cell maintenance and function; these include immunophenotyping, cell cycle and proliferation markers, indicators of cell injury and death, intracellular functional and biochemical analyses [64].

Immunomics involves the integration of the immune-related genomics and proteomics; this approach will help in the synthesis of vast, and sometimes redundant, information. It is of particular relevance to the field of environmental health research in which biological data is collected from subjects to evaluate the associations between environmental exposures (e.g., xenobiotics, allergens) and disease outcomes (e.g., asthma, COPD). For example, single nucleotide polymorphisms (SNPs) in immune-related genes suspected to be involved in disease pathology can be evaluated together with the protein expression of that gene. As it is unlikely that a complete data set of cytokines, for example, can be gathered from one study, inputing the limited data set into an interactive map of the cytokine pathway could prove useful for interpreting the net immune response. This would be particularly useful in instances where there is redundancy or overlap in the functions of cytokines and/or immune cells. Diaz-Ramos *et al.* (2010) [65] recently described the development of a comprehensive immunome that identified 1,015 genes expressed in immune cells or lymphoid tissues that correspond to proteins located on the plasma membrane. The identification of an immunomic profile will contribute to the compilation of “fingerprints” of dysregulated immunity; these will prove useful in the investigation of environmental health diseases and the process of linking environmental exposures to immune disorders.

## 6. Discussion and Conclusions

Immunoinformatics, including the software and hardware capable of synthesizing this information will enable researchers to visualize global changes in protein expression profiles relevant markers of interest [66–68]. Research areas of immunoinformatics include (1) allergy prediction, (2) understanding of immune-related genes, (3) study of genes and their expressions in healthy and diseased states, (4) T- and B- cell epitope prediction, and *in silico* vaccination [66]. Yan (2010) [69] has summarized the resources available on the genetic variation on the immune system. Integration of accumulating data will be an important step in identifying a useful immunologic marker. This can be accomplished by an integrated evaluation of multiple data sets obtained (biological, epidemiological, statistical, clinical trial) and evaluating the risk-benefit evidence. [70]. For example, the use of a scale to rate the level of evidence provided (study design, target outcome, and statistical evaluation), with level 1 the strongest evidence and 5 the weakest; 2 represents a potential surrogate marker. The criteria listed in this rating system were used to evaluate biomarkers for the immune disorder rheumatoid arthritis and the marker CD68, specific for macrophages, was designated a level three (epidemiology studies were not considered to be statistically powered), whereas the soluble marker C-reactive protein was deemed difficult to rank [71]. Weak clinical study design, including power and duration, was cited as the main limitation of this study [72]. Several factors that contribute to the variability of immune parameters are host factors and assay variation, and both of these can be addressed when designing the study. Host factors (e.g., sex, age, ethnicity/geography, nutrition) [73] and exposure factors (e.g., chemicals, bioaerosols, season, smoking, alcohol *etc.*) and disease states (e.g. leukemia, asthma, infections, *etc.*) also contribute to the variability of immune biomarkers [74,75]. For example, when lymphocyte subsets were analyzed in children who ranged in age from newborns to 18 years old, age was found to be an important factor in distributions of cell types [76]. Stress [77] and socioeconomic status [78] also impact the status of asthma in children and adolescents, identified by changes in cytokine biomarkers (IL-4, IL-5, and IFN-γ). In adults, alcohol intake has been associated with increased serum IgE levels [41]. The intra-individual variability should be low, compared to the inter-individual variability. Additionally, the effect of other host factors such as age, gender, stress, exercise, and smoking on biomarker measurement should also be well-characterized. The markers should be analytically detectable and reproducible in the same laboratory and in others.

In conclusion, the use of immune biomarkers in human clinical trials and molecular epidemiology of environmental health can facilitate a better understanding of the mechanisms that underlie associations between environmental exposures and immune-mediated disorders, such as cancer, asthma, and autoimmune disorders. An integrated approach that incorporates host and environmental factors will be particularly important in the development and application of immunologic biomarkers in public health research.

## Figures and Tables

**Table 1.** Biomarkers used to investigate immunotoxicity in human studies.

**Immune Markers**	**Examples of Endpoints**	**Biological Samples**
Cellular phenotype; activation markers	CD3, CD4, CD8, CD11c, CD19, CD25, CD56, CD14, basophils, neutrophils; Activation markers: CD69, CD45RO, CD45RA	Whole blood, Urine
Antibodies	IgM, IgD, IgG, IgA, IgE,	Plasma, Breastmilk
Cytokines	IL-2, IL-4, IL-5, IL-10, IL-13, IFN-γ, TNF-α, GM-CSF	Serum/plasma, peripheral blood, urine, saliva
Chemokines	RANTES, IP-10, MIP-1α, MIP-1β, MDC, TARC	Serum/plasma
Proliferation Tests	Mitogenic stimulation (PHA, Concavalin A, specific antigen)	Peripheral blood

**Table 2.** Findings from selected biomarker studies on the relationship between environmental exposures and health outcomes.

	**Description of Epidemiology Study Design & Subjects**	**Method of exposure assessment**	**Biological sample and (immune biomarkers employed)**	**Key findings and evaluation of concordance**
Atopy	Longitudinal/Prospective; (n = 3,062), combined birth cohorts (ages 1–8 years)	Questionnaire; indoor environment, pet exposure	Peripheral blood (total and specific IgE and CD14/IL13 genotypes)	Atopy influenced by IL13 in <8 years and CD14 with pet interaction in ages 4 and 8 (Bottema *et al*. 2008) [37]
	Longitudinal; birth cohort (n = 172)		Peripheral blood (differential cell counts and IFN-γ, TNF-α, IL-4, IL-5, IL-9, Il-10, IL-13 by ELISA)	Atopy associated with increased Th2; bronchial hyperresponsiveness associated with Th1 (Heaton *et al*. 2005) [38]
	Cross-sectional; children ages 6–16 (n = 24) *vs*. reference group	Questionnaire; parental tobacco smoke	Nasopharangeal aspirate (analyzed for IL-13 cytokine levels)	ETS augments secretion of IL-13 (Feleszko *et al*. 2006) [39]
Asthma	Longitudinal/Prospective; birth cohort (n = 239)	Questionnaire; pesticide and allergen exposures	Peripheral blood (intracellular IFN-γ and IL-4 in T-helper cells)	Th2 cells associated with asthma and wheeze; Th1 associated with breastfeeding and parental occupation in agriculture (Duramad *et al*. 2006) [40]
Asthma	Cross-sectional; children with asthma (n = 33) *vs*. health controls	Questionnaire	Exhaled breath condensate (IFN-γ, TNF-α, IL-2, IL-4, IL-5, IL-10)	Cytokine levels low but detectable; processing method needs improvement (Robroeks *et al*. 2006) [22]
	Case-control retrospective; adults ages 20–79 (n = 3,443)	Questionnaire and blood evaluation: ethanol levels, CDT1, GGT, ASAT, ALAT	Peripheral Blood; (serum IgE)	Positive associations between alcohol consumption and total IgE serum levels in atopic subjects (Friedrich *et al*. 2008) [41]
Chronic Obstructive Pulmonary Disease (COPD)	Cross-sectional; patients with COPD (n = 35), non-smokers (n = 18), and smokers (n = 20)	Questionnaire; criteria for non-smokers was normal spirometry results	Induced sputum (differential cell counts; CXCL9, CXCL10, CXCL11, and CCL5 by ELISA)	CXCR3 and CCL5 increased in COPD patients compared with non smokers (Costa, *et al*. 2008) [42]
	Cross-sectional; patients with COPD (n = 26), smokers (n = 19), healthy non-smokers (n = 5)	Questionnaire; history of smoking	Bronchial Alveolar Lavage (BAL) and peripheral blood (CD3, CD4, CD8, CD45RA, CD25, CD69)	Increased CD8 and CD4+CD25+ in COPD BAL samples (Smyth *et al*. 2007) [43]
	Cross-sectional; COPD (n = 30), divided into two categories: Forced-expiratory volume in 1 second (FEV1) <50% and >50%	Questionnaire; smoking status	Induced sputum (IL-6, IL-8 and TNF-α)	Mean levels of three cytokines elevated in severe *vs*. moderate COPD (Hacievliyagil *et al*. 2005) [44]

1 carbohydrate-deficient transferring (CDT), gamma-glutamyl transferase (GGT), aspartate-amino transferase (ASAT), alanine-amino transferase (ALAT) are biomarkers of recent and long-term exposure to alcohol [45].
